# Antibacterial Mechanisms and Skin-Protective Activities of *Syzygium nervosum* Leaf Extracts from Different Geographical Origins

**DOI:** 10.3390/ijms27146515

**Published:** 2026-07-22

**Authors:** Krissana Khoothiam, Pantira Tachit, Natthaphon Thatsanasuwan, Orada Chumphukam, Saranya Chaiwaree, Nittiya Suwannasom, Thida Kaewkod, Aussara Panya, Ratchanaporn Chokchaisiri, Chutamas Thepmalee

**Affiliations:** 1 Unit of Excellence on Research and Development of Cancer Therapy, University of Phayao, Phayao 56000, Thailand; krissana.kh@up.ac.th (K.K.); nittiya.su@up.ac.th (N.S.); 2 Division of Microbiology, School of Medical Sciences, University of Phayao, Phayao 56000, Thailand; 3 Division of Biochemistry, School of Medical Sciences, University of Phayao, Phayao 56000, Thailand; phungptr2002@icloud.com (P.T.); orada.ch@up.ac.th (O.C.); 4 Division of Nutrition and Dietetics, School of Medical Sciences, University of Phayao, Phayao 56000, Thailand; natthaphon.th@up.ac.th; 5 Department of Pharmaceutical Technology and Biotechnology, Faculty of Pharmacy, Payap University, Chiang Mai 50000, Thailand; saranya_c@payap.ac.th; 6 Department of Biology, Faculty of Science, Chiang Mai University, Chiang Mai 50200, Thailand; thida.kaewkod@cmu.ac.th (T.K.); aussara.pan@cmu.ac.th (A.P.); 7 Cell Engineering for Cancer Therapy Research Group, Department of Biology, Faculty of Science, Chiang Mai University, Chiang Mai 50200, Thailand; 8 Division of Chemistry, School of Science, University of Phayao, Phayao 56000, Thailand; ratchanaporn.ch@up.ac.th

**Keywords:** *Syzygium nervosum*, skin pathogenic bacteria, antibacterial activity, antioxidant activity, anti-tyrosinase activity

## Abstract

Human skin is continuously exposed to microbial invasion and oxidative stress, highlighting the need for safe and effective natural skin-protective agents. This study aims to investigate the antibacterial, antioxidant, and anti-tyrosinase activities of methanolic leaf extracts of *Syzygium nervosum* collected from Chiang Mai (SNLM-CM) and Phayao (SNLM-PY), Thailand. LC–QTOF–MS analysis revealed distinct phytochemical profiles between the extracts, suggesting that geographical origin influenced their biological activities. Antibacterial activity against *Staphylococcus aureus*, *Staphylococcus epidermidis*, *Pseudomonas aeruginosa*, and *Escherichia coli* was evaluated, together with studies of bacterial membrane integrity. Antioxidant capacity was assessed using ABTS, DPPH, FRAP, and ORAC assays, while anti-tyrosinase activity and cytotoxicity toward normal human dermal fibroblasts (NHDF) were determined by MTT assay. Both extracts exhibited potent antibacterial activity, particularly against Gram-positive bacteria (*S. aureus* and *S. epidermidis*) likely through membrane disruption, as evidenced by propidium iodide uptake, intracellular DNA and protein leakage, and scanning electron microscopy observations. SNLM-CM showed stronger antioxidant activity, whereas SNLM-PY demonstrated greater anti-tyrosinase activity. The extracts maintained high NHDF viability at concentrations below 100 µg/mL and retained antibacterial efficacy when incorporated into a cleansing gel formulation. Overall, *S. nervosum* leaf extracts exhibit promising antibacterial, antioxidant, and anti-tyrosinase activities, warranting further investigation to optimize their safety and efficacy for dermatological and cosmetic use.

## 1. Introduction

Human skin is an essential protective barrier between the body and the external environment, providing defense against physical and chemical insults while preventing microbial invasion [1]. Disruption of this barrier can facilitate colonization by opportunistic and pathogenic microorganisms, particularly bacteria responsible for skin and soft tissue infections that may lead to cellular and tissue damage [2]. Among skin-associated bacteria, Gram-positive cocci such as *Staphylococcus aureus* and *Staphylococcus epidermidis* are of particular clinical relevance, causing infections ranging from mild superficial lesions to severe invasive disease, especially in immunocompromised individuals [3,4]. The increasing global spread of antimicrobial resistance has further complicated treatment strategies by reducing the effectiveness of conventional antibiotics [5]. In addition, oxidative stress plays an important role in skin aging and inflammation, where excessive reactive oxygen species contribute to tissue injury and disease progression, highlighting the need for alternative agents with both antimicrobial and antioxidant properties [6].

*Syzygium nervosum*, locally known in Northern Thailand as Ma-kiang, belongs to the Myrtaceae family and is widely distributed throughout Southeast Asia, where it has long been recognized as an important medicinal and edible plant [7]. In traditional medicine, *S. nervosum* has been used for treating gastrointestinal disorders, diarrhea, dysentery, fever, and inflammatory conditions. Its leaves and flower buds are commonly consumed as herbal teas in Vietnam and China, while its fruits are processed into value-added products, such as wines, jellies, and jams, in Northern Thailand, thereby supporting local communities [8]. Recent studies have shown increasing interest in non-fruit parts of *S. nervosum*, particularly the leaves, owing to their abundance of bioactive phytochemicals and potential health-promoting properties [9]. Extracts from Syzygium species have been reported to contain abundant phenolic compounds and flavonoids, which are associated with antioxidant and antibacterial activities. These compounds, particularly flavonoids and phenolic tannins, are considered key contributors to bioactivity, often showing positive correlations with antimicrobial and antioxidant potential [9,10]. Consequently, such phytochemical-rich extracts are increasingly investigated as functional ingredients for herbal and cosmetic applications.

Despite growing evidence supporting the antimicrobial and antioxidant potential of *S. nervosum*, most studies have focused primarily on *S. aureus*, while other clinically relevant skin-associated bacteria remain less explored [7]. In particular, *S. epidermidis*, although a dominant member of the normal skin microbiota, may act as an opportunistic pathogen when skin integrity is compromised [1]. Moreover, the frequent co-colonization and interactions between *S. aureus* and *S. epidermidis* may influence pathogenicity and disease outcomes [11].

Environmental conditions are key factors influencing plant growth, adaptation, and the accumulation of specialized metabolites, which may subsequently affect the phytochemical composition and biological activities of medicinal plants [12]. Although Chiang Mai and Phayao provinces are both located in northern Thailand, they differ in several ecological characteristics, including altitude, average temperature, rainfall patterns, relative humidity, soil properties, and local microclimates. Thus, comparing *S. nervosum* from these two regions provides insight into how geographical origin influences its bioactive properties. Based on this rationale, the present study aimed to evaluate the antibacterial activity of *S. nervosum* leaf extracts collected from different regions of Northern Thailand against skin-associated pathogenic bacteria, particularly *S. aureus* and *S. epidermidis*, and to investigate their antioxidant and anti-tyrosinase activities. In addition, the antibacterial efficacy of SNLM extracts incorporated into a cleansing gel formulation was evaluated to assess the biological activity of the bioactive compounds within the formulation matrix. The findings provide biologically relevant evidence supporting the potential application of Ma-kiang leaf extracts as natural agents for skin-related and cosmetic use.

## 2. Results

### 2.1. Yield of Extraction, Total Phenolic, and Flavonoid Contents of S. nervosum Leaf Methanolic Extracts

The extraction yield, total phenolic content (TPC), and total flavonoid content (TFC) of methanolic leaf extracts of *S. nervosum* collected from Chiang Mai (SNLM-CM) and Phayao (SNLM-PY), Thailand, were assessed and presented in Table 1. SNLM-PY showed a higher extraction yield (6.44%) than SNLM-CM (4.55%). Despite a lower initial sample weight, SNLM-PY produced a greater extract weight, indicating higher extraction efficiency. The total phenolic content (TPC) of SNLM-PY was significantly higher than that of SNLM-CM (179.82 ± 6.26 and 167.48 ± 3.66 mg GAE/g sample, respectively; *p* < 0.05), indicating differences in phenolic accumulation between samples collected from different geographical regions. In contrast, total flavonoid content was not detected in either extract under the experimental conditions used. Overall, the results suggest that geographical origin may influence the extraction yield and phenolic content of *S. nervosum* leaf extracts, with the Phayao-derived extract showing slightly higher yield and phenolic content than the Chiang Mai-derived extract.

### 2.2. LC–QTOF–MS Phytochemical Profiling of SNLM Extracts

The phytochemical composition of SNLM extracts was analyzed by LC–QTOF–MS, and compound identification was tentatively assigned based on retention times, molecular masses, expected *m*/*z* values, and comparisons with previous reports. As summarized in Table 2, a total of 20 phytochemicals belonging to phenolic acids, flavonoids, terpenoids, and polyphenolic derivatives were detected in both SNLM-CM and SNLM-PY extracts. Several identified compounds, including gallic acid, reynoutrin, eugenyl acetate, arjunolic acid, cleistocaltone B, and ursolic acid, have been reported to possess antioxidant and antimicrobial activities [13,14,15,16,17,18], suggesting their contribution to the biological effects observed in this study. Overall, LC–QTOF–MS profiling demonstrated that SNLM extracts contain diverse bioactive phytochemicals associated with antioxidant and antibacterial properties.

As shown in Table S1, the phytochemical compounds tentatively identified in SNLM-CM included flavonoids, flavanones, chalcone derivatives, phenolic acids, and triterpenoids. Notable compounds detected in SNLM-CM included quercetin, kaempferol, tamarixetin, phloretin, mearnsitrin, ellagic acid, and several methoxylated chalcone and flavanone derivatives. In contrast, compounds were identified in SNLM-PY (Table S2), comprising polyphenols, flavonoid glycosides, galloylated compounds, terpenoids, and phenolic acids. Major constituents included (-)-epigallocatechin 3-O-gallate, myricetin-3-O-glucuronide, myricetin-3-O-glucoside, 1,2,3,4,6-penta-O-galloyl-β-D-glucose, cinnamic acid, lupeol, and jambone F. These findings demonstrate that the phytochemical composition of *S. nervosum* leaves varies according to geographical origin. SNLM-CM was characterized by the presence of diverse flavonoid and chalcone derivatives, whereas SNLM-PY contained a wider range of polyphenolic and galloylated compounds. Given these compositional differences, the antibacterial, antioxidant, and anti-tyrosinase activities of the SNLM extracts were subsequently evaluated.

### 2.3. Antibacterial Activity of S. nervosum Leaf Extracts Against Selected Gram-Positive and Gram-Negative Bacterial Strains

The antibacterial activities of SNLM-CM and SNLM-PY against *S. aureus*, *S. epidermidis*, *E. coli*, and *P. aeruginosa* were evaluated. As shown in Table 3, both methanolic leaf extracts of *S. nervosum* exhibited antibacterial activity against the tested bacteria. Against *S. aureus*, SNLM-CM showed stronger activity than SNLM-PY, with a larger inhibition zone (20.00 ± 2.00 vs. 11.67 ± 0.58 mm) and lower MIC/MBC values (3.26/6.52 vs. 6.52/13.03 mg/mL). For *S. epidermidis*, SNLM-CM produced a larger inhibition zone (23.33 ± 2.08 mm) than SNLM-PY (17.67 ± 1.53 mm), although SNLM-PY showed lower MIC and MBC values (3.26 and 6.52 mg/mL) compared with SNLM-CM (5.21 and 9.12 mg/mL). Both extracts showed weaker activity against Gram-negative bacteria. For *E. coli*, inhibition zones were 7.00 ± 1.73 mm (SNLM-CM) and 6.17 ± 0.29 mm (SNLM-PY), with MIC values of 15.63 and 31.25 mg/mL, respectively. Against *P. aeruginosa*, SNLM-CM exhibited greater inhibition (14.33 ± 1.15 mm) than SNLM-PY (9.67 ± 1.15 mm), with MIC values of 10.42 and 15.63 mg/mL, respectively. Overall, both extracts showed greater antibacterial activity against Gram-positive bacteria, with SNLM-CM exhibiting stronger effects than SNLM-PY. Therefore, Gram-positive skin-pathogenic bacteria, including *S. aureus* and *S. epidermidis*, were selected for subsequent investigation of the antibacterial mechanism of SNLM extracts.

### 2.4. Time–Kill Kinetics of S. nervosum Leaf Extracts Against Skin Pathogenic Bacteria

**Figure 1:**
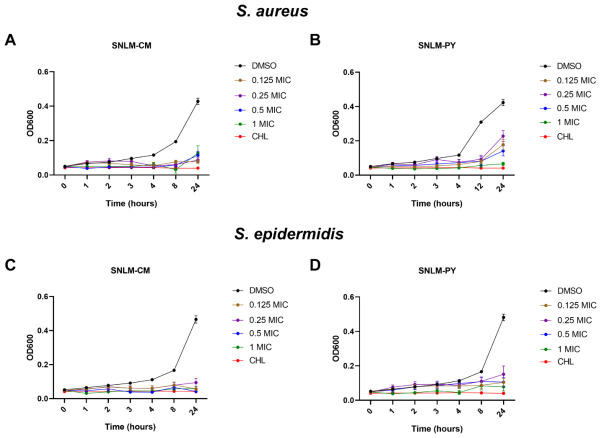
Time–kill kinetics of skin pathogenic bacteria. SNLM-CM and SNLM-PY extracts at concentrations of 0.125×–1× MIC were used to treat *S. aureus* and *S. epidermidis* for 0, 1, 2, 3, 4, 8, and 24 h. DMSO was employed as the negative control, whereas chloramphenicol served as the positive control. The bacterial growth curves of *S. aureus* (**A**–**D**) after treatment, measured by spectrophotometry (OD at 600 nm). Results are presented as the mean ± SEM from three independent experiments (N = 3).

To explore the pharmacological activity of the SNLM extracts, we investigated the bactericidal effects on the growth kinetics of *S. aureus* and *S. epidermidis* following treatment with SNLM-CM, SNLM-PY, chloramphenicol, and diluent control (DMSO). The time–kill kinetics of methanolic leaf extracts of *S. nervosum* from Chiang Mai (SNLM-CM) and Phayao (SNLM-PY) against *S. aureus* and *S. epidermidis* are shown in Figure 1. In the untreated control (DMSO), bacterial growth increased progressively over time, reaching the highest optical density at 24 h. In contrast, treatment with SNLM extracts resulted in dose-dependent suppression of bacterial growth. At concentrations of 0.125–0.5× MIC, bacterial growth was partially reduced, whereas treatment at 1× MIC maintained low OD600 values throughout the incubation period, comparable to the chloramphenicol control. Both SNLM-CM and SNLM-PY showed similar inhibitory trends, although SNLM-CM generally produced slightly stronger growth suppression, particularly against *S. aureus*. Overall, the results indicate that the extracts effectively inhibited bacterial growth over time, with stronger effects observed at higher concentrations.

### 2.5. Bacterial Cell Viability of Skin Pathogenic Bacteria After SNLM Extract Treatment

**Figure 2:**
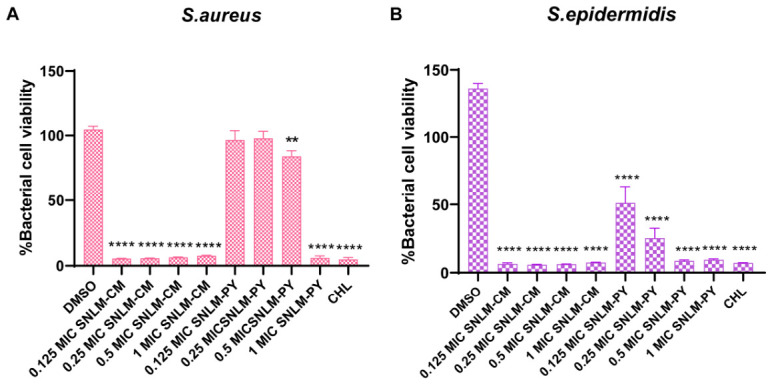
Bacterial cell viability of *S. aureus* and *S. epidermidis* after treatment with SNLM extracts. *S. aureus* and *S. epidermidis* were incubated with SNLM-CM (**A**) and SNLM-PY (**B**) at 0.125×, 0.25×, 0.5×, and 1× MIC, DMSO, and chloramphenicol (CHL) for 24 h. Bacterial cell viability was assessed using PrestoBlue Reagent. Results are presented as the mean ± SEM from three independent experiments (N = 3). Significant differences between DMSO controls and tested conditions were analyzed using one-way ANOVA and are indicated by asterisks (** *p* < 0.01 and **** *p* < 0.001).

Next, the effectiveness of the SNLM extracts against *S. aureus* and *S. epidermidis* was confirmed using a Resazurin-based viability assay. As shown in Figure 2, treatment with SNLM extracts significantly reduced the viability of *S. aureus* and *S. epidermidis* after 24 h compared with the DMSO control. For *S. aureus*, SNLM-CM markedly decreased cell viability across all tested concentrations (0.125–1× MIC; *p* < 0.001, IC_50_ < 0.41 mg/mL), indicating strong inhibition comparable to the chloramphenicol control, whereas SNLM-PY showed a significant reduction primarily at 1× MIC, with IC_50_ = 3.19 ± 0.34 mg/mL. In *S. epidermidis*, both extracts significantly reduced bacterial viability across all concentrations (*p* < 0.001), with SNLM-CM showing an IC_50_ < 0.65 mg/mL and SNLM-PY an IC_50_ of 0.29 ± 0.06 mg/mL. Overall, the results confirm that the SNLM extracts reduced bacterial viability in a concentration-dependent manner.

### 2.6. Effect of SNLM Extracts on Bacterial Membrane Integrity

**Figure 3:**
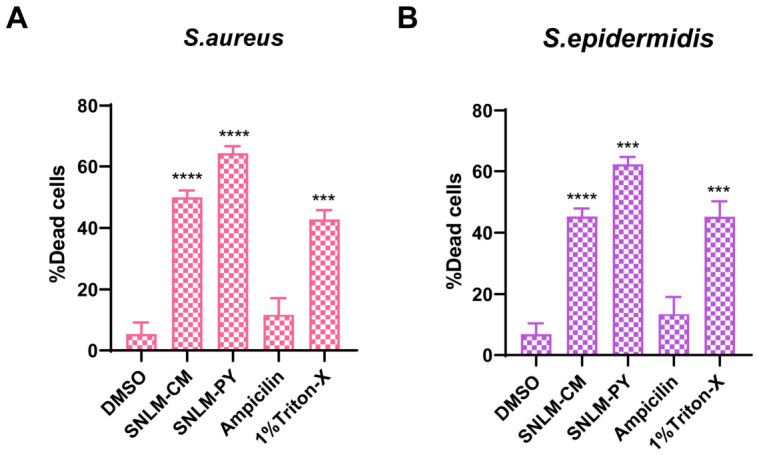
Effect of SNLM extracts on bacterial membrane integrity. *S. aureus* (**A**) and *S. epidermidis* (**B**) were exposed to 1× MIC of SNLM extracts, DMSO, ampicillin, or 1% Triton-X at 37 °C for 1 h. The percentages of dead bacterial cells were evaluated by dual-fluorescence staining for membrane integrity using the bacterial viability assay kit. Results are presented as the mean ± SEM from three independent experiments (N = 3). Significant differences between DMSO controls and tested conditions were analyzed using one-way ANOVA and are indicated by asterisks (*** *p* < 0.001 and **** *p* < 0.0001).

Bacterial membrane integrity was next evaluated by dual-fluorescence staining, using the Bacterial Viability Assay following treatment with SNLM extracts, ampicillin, or 1% Triton-X. Both SNLM extracts significantly increased membrane permeability in *S. aureus* and *S. epidermidis* compared with the DMSO control, as indicated in Figure 3. In *S. aureus*, treatment with SNLM-CM and SNLM-PY significantly increased the percentage of dead cells to approximately 50.00 ± 2.25% (*p* < 0.0001) and 64.56 ± 2.28% (*p* < 0.0001), respectively, compared with the DMSO control. Similar effects were observed in *S. epidermidis*, in which the percentages of dead cells increased to approximately 45.32 ± 2.59% (*p* < 0.001) and 62.33 ± 2.55% (*p* < 0.0001), respectively, compared with the DMSO control. These values were markedly higher than those of the negative control and comparable to those of the positive membrane-disrupting control (1% Triton-X). Ampicillin was included as an antibiotic control to represent a conventional cell wall-targeting agent; it caused less membrane damage than the SNLM extracts, suggesting that the extracts may exert stronger membrane-associated effects. Overall, the results suggest that the antibacterial activity of SNLM extracts is associated with increased membrane permeability, leading to bacterial cell death.

### 2.7. Effect of SNLM Extracts on DNA and Protein Release

**Figure 4:**
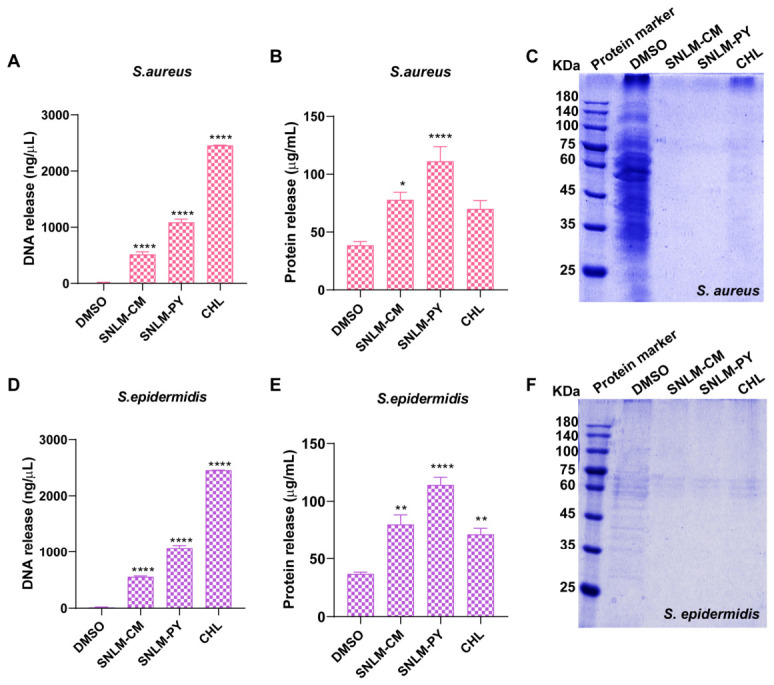
Effect of SNLM extracts on DNA and protein release from *S. aureus* and *S. epidermidis*. Leakage of nucleotides and proteins from bacterial cells following treatment with 1× MIC of SNLM extracts, DMSO, and chloramphenicol for 3 h at 37 °C. The DNA and protein contents of bacterial cells in culture supernatants were evaluated using a microspectrophotometer at 260 nm (**A**,**D**) and the Bradford assay (**B**,**E**), respectively. The remaining bacterial cells, *S. aureus* (**C**) and *S. epidermidis* (**F**), were lysed with the lysis buffer. The bacterial protein lysate was subjected to SDS-PAGE and stained with Coomassie brilliant blue. Results are presented as the mean ± SEM from three independent experiments (N = 3). Significant differences between DMSO controls and the tested conditions were analyzed using one-way ANOVA and are indicated by asterisks (* *p* < 0.05, ** *p* < 0.01, and **** *p* < 0.001).

To evaluate membrane damage induced by SNLM extracts, the leakage of intracellular DNA and proteins of *S. aureus* and *S. epidermidis* was determined by measuring absorbance at 260 nm and using the Bradford protein assay, respectively. Figure 4A,B (*S. aureus*) shows that treatment with SNLM-CM and SNLM-PY extracts significantly increased both DNA (515.8 ± 45.47 ng/µL; *p* < 0.0001 and 1086 ± 58.39 ng/µL; *p* < 0.0001) and protein release (77.88 ± 6.59 µg/mL; *p* < 0.05 and 111.1 ± 12.67 µg/mL; *p* < 0.0001) compared with DMSO control (9.79 ± 9.79 ng/µL and 38.63 ± 3.25 µg/mL), indicating disruption of cellular integrity. SNLM-PY induced higher DNA and protein leakage than SNLM-CM, while the chloramphenicol (CHL) group exhibited the highest DNA release. SDS-PAGE analysis (Figure 4C) further supported these findings, as strong protein bands observed in the control were markedly reduced in SNLM- and CHL-treated groups, suggesting leakage or loss of intracellular proteins following treatment. Figure 4D,E (*S. epidermidis*) shows a similar trend, with both SNLM-CM and SNLM-PY extracts significantly increasing extracellular DNA (555.60 ± 15.85 ng/µL; *p* < 0.0001 and 1072 ± 44.06 ng/µL; *p* < 0.0001) and protein levels (79.88 ± 8.479 µg/mL; *p* < 0.01 and 114.10 ± 6.72 µg/mL; *p* < 0.0001) relative to the control (13.22 ± 7.22 ng/µL for DNA leakage and 36.75 ± 1.61 µg/mL). SNLM-PY again showed greater leakage compared with SNLM-CM, whereas CHL produced the strongest DNA release. Then, SDS-PAGE profiles of *S. epidermidis* (Figure 4F) revealed reduced protein band intensity in treated samples compared with the DMSO group, strongly confirming that exposure to SNLM extracts caused cellular damage leading to the release of intracellular components.

### 2.8. Effect of SNLM Extracts on Bacterial Cell Structure and Morphology

**Figure 5:**
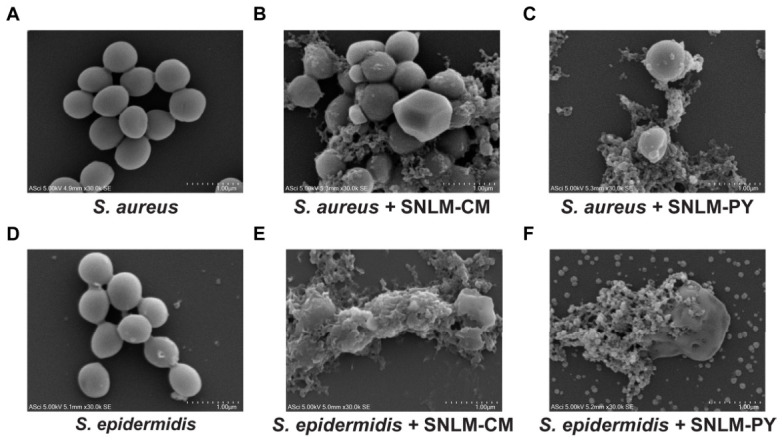
Effect of SNLM extracts on bacterial cell morphology. *S. aureus* and *S. epidermidis* were exposed to either SNLM-CM or SNLM-PY at 1× MIC for 3 h at 37 °C. Scanning electron microscopy (SEM) images acquired at 30,000× magnification show (**A**) untreated *S. aureus*; (**B**) *S. aureus* treated with SNLM-CM; (**C**) *S. aureus* treated with SNLM-PY; (**D**) untreated *S. epidermidis*; (**E**) *S. epidermidis* treated with SNLM-CM; and (**F**) *S. epidermidis* treated with SNLM-PY. Scale bar = 1.00 µm.

Scanning electron microscopy (SEM) was used to evaluate bacterial cell structure and morphological changes after treatment with SNLM extracts at 1× MIC for 2 h at 37 °C. Figure 5 shows the morphological characteristics of *S. aureus* and *S. epidermidis* before and after exposure to SNLM-CM and SNLM-PY. Untreated bacterial cells exhibited a typical spherical morphology with smooth, intact surfaces and clearly defined cell boundaries (Figure 5A,D). For *S. aureus*, treatment with SNLM-CM (Figure 5B) and SNLM-PY (Figure 5C) resulted in severe structural alterations characterized by irregular cell morphology, disrupted cell surfaces, and accumulation of damaged cellular debris. The SNLM-PY-treated cells exhibited more extensive deformation and a loss of the typical coccal structure. Similarly, *S. epidermidis* exposed to SNLM-CM (Figure 5E) and SNLM-PY (Figure 5F) exhibited pronounced morphological changes, including collapsed cell structures, roughened surfaces, aggregation of cellular fragments, and disruption of normal cell architecture compared with untreated cells. These observations suggest that SNLM extracts interfere with cell membrane integrity, leading to morphological damage and potential leakage of intracellular contents, which may contribute to bacterial cell death.

### 2.9. Antioxidant and Anti-Tyrosinase Activities of SNLM Extracts

SNLM extracts were evaluated for their antioxidant and anti-tyrosinase activities. The antioxidant and anti-tyrosinase activities of SNLM extracts are presented in Table 4. Both SNLM-CM and SNLM-PY exhibited notable antioxidant activity, although differences in potency were observed across assays. In the ABTS assay, SNLM-CM exhibited potent radical-scavenging activity, with an IC_50_ value of 8.00 ± 2.00 µg/mL, whereas SNLM-PY showed an IC_50_ value of 11.00 ± 1.44 µg/mL. Compared with the reference antioxidant Trolox (IC_50_ = 6.95 ± 0.24 µg/mL), SNLM-CM exhibited comparable antioxidant activity, whereas SNLM-PY showed significantly lower activity (*p* < 0.05). In the DPPH assay, SNLM-PY displayed a lower IC_50_ value (11.00 ± 1.12 µg/mL) than SNLM-CM (16.00 ± 1.79 µg/mL), indicating greater DPPH radical-scavenging potential. However, both extracts exhibited significantly lower antioxidant activity than Trolox (IC_50_ = 6.40 ± 0.05 µg/mL; *p* < 0.01 and *p* < 0.001, respectively). In reducing power assays, SNLM-CM exhibited higher FRAP (1525.71 ± 24.62 mM TE/g sample) and ORAC values (997.05 ± 40.80 µM TE/mg sample) than SNLM-PY (1306.79 ± 49.75 mM TE/g sample and 750.29 ± 22.50 µM TE/mg sample, respectively). Regarding anti-tyrosinase activity, SNLM-PY exhibited stronger inhibition with a lower IC_50_ (1.28 ± 0.28 mg/mL), whereas SNLM-CM showed a higher IC_50_ (5.61 ± 0.24 mg/mL). However, both extracts were less potent than the positive control, kojic acid, which showed an IC_50_ value of 0.118 ± 0.01 mg/mL. Overall, both extracts demonstrated antioxidant and anti-tyrosinase potential, with activity profiles that varied across assay systems.

### 2.10. Cytotoxicity of SNLM Extracts Toward Normal Human Dermal Fibroblasts

**Figure 6:**
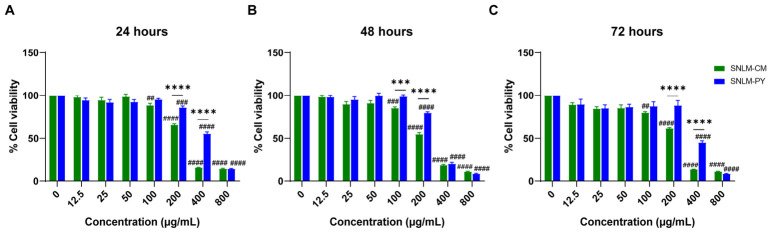
Cytotoxicity of SNLM extracts against normal human dermal fibroblast (NHDF). NHDF cells were treated with SNLM-CM and SNLM-PY extracts at concentrations of 12.5–800 µg/mL for 24 h (**A**), 48 h (**B**), and 72 h (**C**). Cell viability was determined using the MTT assay. Data are presented as mean ± SEM from three independent experiments (N = 3). Statistical significance was analyzed by two-way ANOVA. The difference between the treated group and the DMSO control (0 µg/mL) is indicated by hash symbols (## *p* < 0.01, ### *p* < 0.001 and #### *p* < 0.0001), whereas differences between SNLM-CM and SNLM-PY at the same concentration and time point are indicated by asterisks (*** *p* < 0.001 and **** *p* < 0.0001).

The cytotoxic effects of SNLM-CM and SNLM-PY on normal human dermal fibroblasts (NHDF) were evaluated after 24, 48, and 72 h of exposure using the MTT assay (Figure 6). Overall, both extracts maintained high cell viability at low concentrations (12.5–50 µg/mL), with viability values remaining close to the untreated control across all incubation periods. At 24 h, SNLM-CM slightly reduced NHDF viability at 100 µg/mL, showing a significant decrease compared with the untreated control (*p* < 0.05), and markedly decreased viability to approximately 65.69 ± 1.24% at 200 µg/mL (*p* < 0.0001). In contrast, SNLM-PY showed lower cytotoxicity, maintaining cell viability above 80% even at 200 µg/mL. However, a significant difference between SNLM-CM and SNLM-PY was observed at this concentration (*p* < 0.0001). Similar trends were observed at 48 and 72 h, where SNLM-CM induced a concentration-dependent reduction in viability, particularly at 100 and 200 µg/mL. At 48 h, SNLM-CM significantly reduced cell viability at 100 µg/mL (*p* < 0.05) and 200 µg/mL (*p* < 0.0001), whereas SNLM-PY maintained significantly higher viability (80.46 ± 1.41%) at 200 µg/mL compared with SNLM-CM (57.27 ± 1.27%; *p* < 0.0001). Likewise, at 72 h, SNLM-CM significantly decreased NHDF viability at 100 µg/mL (80.19 ± 1.29%; *p* < 0.05) and more prominently at 200 µg/mL (61.53 ± 0.89%; *p* < 0.0001). SNLM-PY (88.37 ± 5.58%) exhibited significantly lower cytotoxicity than SNLM-CM (61.53± 0.89%) at 200 µg/mL (*p* < 0.0001). The NHDF IC_50_ values of SNLM-CM were 214 ± 6.43, 188.7 ± 7.58, and 183.0 ± 12.53 µg/mL after 24, 48, and 72 h of exposure, respectively. In contrast, SNLM-PY exhibited higher IC_50_ values of 369.0 ± 15.67, 264.2 ± 7.19, and 345.3 ± 23.68 µg/mL at the corresponding time points, further supporting its lower cytotoxicity toward NHDF cells compared with SNLM-CM.

### 2.11. Antibacterial Potential of SNLM-Based Cleansing Gel Against Skin Pathogenic Bacteria

**Figure 7:**
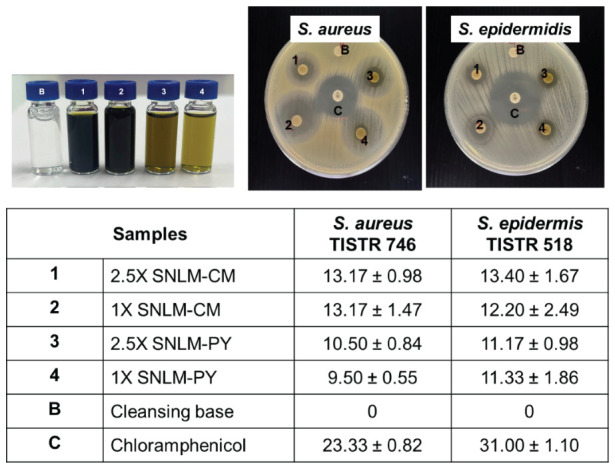
The potential of SNLM extracts for the development of cleansing gel against skin pathogenic bacteria. SNLM extracts were incorporated into a cleansing gel formulation. The antibacterial activity of the cleansing gel formulations against *S. aureus* and *S. epidermidis* was determined by the disc diffusion method. Representative images of the zone of inhibition produced by the formulations against *S. aureus* and *S. epidermidis* are shown. Samples were included: 2.5× SNLM-CM, 1× SNLM-CM, 2.5× SNLM-PY, 1× SNLM-PY, cleansing base, and chloramphenicol as the positive control. The diameters of inhibition zones (mm) are presented as mean ± SEM of three independent experiments (N = 3).

Taken together, *S. nervosum* leaf methanolic extracts (SNLM) collected from different geographical origins exhibited potent multifunctional skin-protective properties, including antibacterial, antioxidant, and anti-tyrosinase activities. Based on these promising bioactivities, a cleansing gel formulation incorporating *S. nervosum* leaf extracts was subsequently developed and evaluated. The antibacterial activity of cleansing gel formulations containing SNLM extracts was evaluated against *S. aureus* and *S. epidermidis*, as shown in Figure 7. Both SNLM-CM and SNLM-PY formulations exhibited inhibitory activity against the tested bacteria, whereas the cleansing base alone showed no inhibitory activity. The 2.5× concentration of SNLM-CM produced inhibition zones of 13.17 ± 0.98 mm against *S. aureus* and 13.40 ± 1.67 mm against *S. epidermidis*, which were slightly higher than those observed for the 1× SNLM-CM formulation. Similarly, SNLM-PY formulations demonstrated antibacterial activity, with inhibition zones ranging from 9.50 ± 0.55 to 11.17 ± 0.98 mm. Overall, SNLM-CM formulations showed stronger antibacterial effects than SNLM-PY. As expected, chloramphenicol produced the largest inhibition zones, serving as a positive control. These results indicate that incorporating SNLM extracts into a cleansing gel formulation retains antibacterial activity against skin-pathogenic bacteria.

## 3. Discussion

The present study shows that *S. nervosum* leaf extracts exhibit antibacterial, antioxidant, and anti-tyrosinase activities relevant to skin health. These findings support the growing use of plant-derived compounds as alternatives to conventional therapies, particularly amid rising antimicrobial resistance and oxidative stress [5,6]. The inhibitory effects of *S. nervosum* leaf extracts against *S. aureus* and *S. epidermidis* are consistent with reports on the susceptibility of Gram-positive bacteria to phenolic- and flavonoid-rich extracts [7,10]. The antioxidant activity further suggests a role in reducing oxidative damage associated with skin inflammation and aging [5,6]. Overall, these results support the potential application of *S. nervosum* leaf extracts in dermatological and cosmetic use.

The comparative analysis showed clear differences between *S. nervosum* leaf extracts from Chiang Mai (SNLM-CM) and Phayao (SNLM-PY). Both extracts contained high levels of phenolics, but SNLM-PY exhibited a higher extraction yield and a slightly higher total phenolic content. These differences are likely due to environmental and geographical factors, such as climate, altitude, and soil conditions, which influence the production of plant secondary metabolites [19,20]. Similar location-dependent variation in phytochemical composition has been reported in other medicinal plants [21,22]. The higher yield and phenolic content in SNLM-PY suggest a greater abundance of extractable polar compounds, particularly phenolics, which are associated with antioxidant and antimicrobial activities [21]. In contrast, flavonoids were not detected in either extract using the aluminum chloride (AlCl_3_) colorimetric assay. This may be attributed to flavonoid concentrations being below the assay detection capability, as the reported limits of detection (LOD) and quantification (LOQ) are 1.47 and 4.90 µg/mL (expressed as quercetin equivalents), respectively. In addition, structurally modified flavonoids, such as glycosylated or methylated derivatives, may exhibit reduced Al^3+^ complex formation, leading to weak colorimetric responses and remaining undetected by this method [23]. Furthermore, variations in flavonoid recovery and abundance may also be influenced by extraction efficiency, solvent polarity, plant origin, and phytochemical characteristics [24]. In contrast, LC–QTOF–MS provides substantially higher sensitivity (LODs of 0.01–0.04 µg/mL and LOQs of 0.02–4 µg/mL) and enables detection of individual flavonoid derivatives at trace levels. Thus, the undetectable TFC values observed in this study do not indicate the absence of flavonoids but rather suggest that these compounds were present at concentrations below the detection capability of the AlCl_3_ assay or possessed structural characteristics that limited complex formation. Overall, these findings indicate that geographical origin affects the biochemical profile of *S. nervosum* leaves and should be considered when evaluating their bioactivity.

The antibacterial activity of both SNLM-CM and SNLM-PY is likely related to their high phenolic content (Table 1). Phenolic compounds are known to inhibit bacteria through multiple mechanisms, including disruption of membrane integrity, increased permeability, and interference with enzymatic activity and biofilm formation [25,26]. Although SNLM-PY had slightly higher total phenolic content, SNLM-CM exhibited stronger antibacterial activity, particularly against *S. aureus* and *P. aeruginosa*. This suggests that activity depends not only on phenolic quantity but also on composition and possible synergistic effects among compounds [25,27]. Both extracts were more effective against Gram-positive than against Gram-negative bacteria, likely due to the outer membrane barrier in Gram-negative cells, which limits compound penetration [26,28]. In addition, geographical origin may influence these differences, as environmental factors affect phenolic biosynthesis and bioactivity [22]. Overall, phenolic-rich extracts of *S. nervosum* contribute to antibacterial activity, and compositional variation likely determines their effectiveness.

The antioxidant activity of SNLM extracts varied across assays, as is common for plant-derived extracts. Differences between ABTS and DPPH assays lie in their reaction mechanisms and solubility; ABTS detects both hydrophilic and lipophilic antioxidants, whereas DPPH is more selective and influenced by steric factors [29,30]. This may explain why SNLM-CM showed stronger ABTS activity, whereas SNLM-PY exhibited slightly higher DPPH scavenging. Higher FRAP and ORAC values in SNLM-CM indicate greater reducing power and peroxyl radical-scavenging ability, typically associated with phenolic compounds [31,32]. In contrast, SNLM-PY exhibited greater anti-tyrosinase activity despite lower antioxidant capacity, suggesting that tyrosinase inhibition is not solely dependent on overall antioxidant potential but rather on the presence of specific bioactive compounds that interact with the enzyme. These interactions may involve copper ion chelation at the tyrosinase catalytic active site and enzyme–inhibitor binding affinity [33]. Certain phenolics or related metabolites may directly interact with the tyrosinase active site or chelate copper ions [34,35]. Overall, these differences likely reflect variation in phytochemical composition influenced by geographical origin, highlighting that antioxidant activity and tyrosinase inhibition are not always directly correlated.

The cytotoxicity results showed a clear dose-dependent effect of SNLM extracts on normal human dermal fibroblast (NHDF) cells, with high viability at low to moderate concentrations (less than 12.5–100 µg/mL) and reduced viability at higher concentrations (more than 100 µg/mL), suggesting that lower concentrations may be suitable for topical formulations. This pattern is typical for plant extracts, where increasing concentrations may induce cellular stress, reduce metabolic activity, and consequently decrease cell viability in a dose-dependent manner [36]. SNLM-PY exhibited lower cytotoxicity than SNLM-CM, as reflected by its higher NHDF viability and IC_50_ values across all exposure times. However, the antibacterial IC_50_ values were generally comparable to or higher than those for the NHDF, indicating limited selectivity of the crude extracts. Nevertheless, both extracts showed measurable antibacterial effects at sub-MICs, suggesting that bacterial growth can be suppressed before complete inhibition is achieved. These findings highlight the antibacterial potential of SNLM extracts and emphasize the need for active compound isolation, formulation optimization, and further skin-compatibility studies to improve their selectivity and suitability for dermatological applications. Importantly, SNLM-PY maintained good viability at low to moderate concentrations, indicating acceptable biocompatibility for normal skin cells. Similar findings have been reported for phenolic-rich extracts, which often show antimicrobial activity with limited toxicity to mammalian cells [25,37].

These results indicate a clear link between the antibacterial mechanism of SNLM extracts and their potential use in topical formulations. Mechanistic assays showed increased membrane permeability, leakage of intracellular components, and structural damage in bacterial cells, suggesting that membrane disruption is a key mode of action. Similar effects have been reported for phenolic-rich plant extracts that target bacterial membranes and reduce viability [25,28]. The antibacterial activity of the SNLM-containing cleansing gel further supports its practical application. The formulation inhibited *S. aureus* and *S. epidermidis*, while the base alone showed no effect, confirming that the activity was due to the extracts. Previous studies have shown that plant-derived compounds can remain active in topical formulations when properly formulated [38]. Although the activity was lower than that of chloramphenicol, the formulation demonstrated meaningful antibacterial effects with a safer profile. Overall, SNLM extracts show potential as multifunctional bioactive ingredients for use in cleansing gels and skin-care applications. LC–QTOF–MS profiling revealed that SNLM extracts contain a diverse range of phenolic acids, flavonoids, and triterpenoid-related compounds, many of which are known for their antioxidant and antimicrobial activities. Compounds such as gallic acid, eugenol, and ellagic acid derivatives can scavenge free radicals through electron donation and metal chelation [25,29]. This supports the strong antioxidant activity observed in the ABTS, DPPH, FRAP, and ORAC assays, suggesting that the effects arise from the combined actions of multiple phytochemicals rather than a single compound. Several identified compounds are also known to disrupt bacterial membranes and cellular processes. Phenolics and terpenoid-like molecules can increase membrane permeability and cause leakage of intracellular contents, consistent with the membrane damage and macromolecule release observed in this study [28]. This agreement between chemical profiling and mechanistic assays indicates that antibacterial activity likely involves multi-target effects driven by the extract composition [26,39].

The multifunctional properties of SNLM extracts suggest potential utility in topical and skin-care formulations. Their antioxidant activity may help mitigate oxidative stress–mediated skin damage. In contrast, their antibacterial activity against *S. aureus* and *S. epidermidis*, two common skin-associated microorganisms, may support skin hygiene and help prevent microbial skin disorders. Furthermore, the favorable cytotoxicity profile observed in NHDF cells indicates acceptable biocompatibility for potential topical application. The retained antibacterial activity in the cleansing gel indicates that SNLM extracts maintained their antibacterial potential after incorporation into the formulation.

Overall, LC–QTOF–MS data support the multifunctional bioactivity of SNLM extracts and their potential as natural ingredients for skin-related applications. This study has some limitations that should be considered. First, the experiments were conducted in vitro, which may not fully capture the complexity of real skin environments. Second, this study relied on crude methanolic extracts, which contain complex mixtures of phytochemicals that may exert combined or synergistic effects. Although LC–QTOF–MS provided an overview of the phytochemical composition, individual compounds were not isolated, purified, or quantitatively analyzed, limiting the identification of specific bioactive constituents responsible for the observed antibacterial, antioxidant, and anti-tyrosinase activities. In addition, cytotoxicity was evaluated using only a single normal human cell line (NHDF). Therefore, further studies employing additional skin-relevant cell types, such as keratinocytes, as well as in vivo safety models, would provide a more comprehensive assessment of the extracts’ biocompatibility and safety. The present study evaluated only the antibacterial activity of the formulated cleansing gel. Although the crude SNLM extracts exhibited antioxidant and anti-tyrosinase activities, these biological properties were not assessed after incorporation into the gel formulation. Moreover, the stability, retention of bioactivity, and long-term performance of SNLM extracts after incorporation into topical formulations were not extensively investigated in the present study. Future research should focus on the isolation and characterization of active constituents, formulation optimization, stability testing, bioactivity retention, and comprehensive topical safety evaluations to better define the therapeutic potential and practical applicability of SNLM extracts for dermatological and cosmetic applications.

## 4. Materials and Methods

### 4.1. Plant Materials

Fully expanded mature leaves of *S. nervosum* were collected during the vegetative growth stage prior to flowering/fruiting in May 2023 from two different geographical locations in northern Thailand: Chiang Mai Province (18°49′27.3″ N 99°07′31.8″ E; approximately 320–350 m above sea level) and Phayao Province (19°01′38.1″ N 99°53′34.9″ E; approximately 450–500 m above sea level). The Chiang Mai collection site is located in a mountainous area characterized by well-drained loamy soils and a tropical savanna climate, with an average annual temperature of approximately 25–27 °C and annual rainfall of 1100–1300 mm. In contrast, the Phayao collection site is situated in a higher-elevation intermontane basin, consisting mainly of loamy to clay loam soils, with slightly cooler climatic conditions (average annual temperature of 24–26 °C) and annual rainfall of approximately 1000–1200 mm. Voucher specimens were deposited at the Herbarium, Faculty of Pharmacy, Chiang Mai University, Thailand, under reference number 0023402 [40].

### 4.2. Preparation of the Methanolic Extract of S. nervosum Leaves (SNLM)

**Figure 8:**
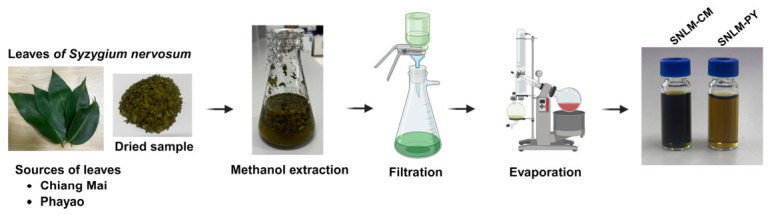
Illustrates the preparation step for SNLM extracts.

Briefly, *S. nervosum* leaves were air-dried at room temperature and ground into a fine powder. The powder was sequentially extracted with absolute methanol (Fisher Chemical, Waltham, MA, USA) at room temperature for 24 h, and the extraction was repeated twice to ensure complete extraction of bioactive constituents. The mixture was then filtered to remove any solid material, followed by evaporation to dryness under reduced pressure at 40–45 °C, yielding SNLM-CM and SNLM-PY, as shown in Figure 8. The dried extracts were reconstituted with dimethyl sulfoxide (DMSO; Sigma-Aldrich, St. Louis, MO, USA) to prepare stock solutions. The mixture was thoroughly vortexed to obtain a homogeneous preparation and filtered through a sterile syringe filter (0.22 µm pore size) (Cytiva, Marlborough, MA, USA) before use.

### 4.3. Determination of the Extraction Yield

The extraction yield of the SNLM was calculated on a dry-weight basis. The yield was determined by comparing the mass of the dried crude extract after solvent removal with the initial dry weight of the plant material used for extraction, following procedures commonly used in phytochemical investigations [24].

### 4.4. Total Phenolic Content (TPC)

The total phenolic content (TPC) of the SNLM extracts was determined by the Folin–Ciocalteu method following a previously reported protocol [40].

### 4.5. Total Flavonoid Content (TFC)

The total flavonoid content (TFC) of the SNLM extracts was determined by the aluminum chloride colorimetric assay following a previously reported protocol [40].

### 4.6. Determination of Phytochemicals of SNLM Extracts by LC–QTOF–MS

The phytochemical constituents of the SNLM extracts obtained from Chaing-Mai (SNLM-CM) and Phayao (SNLM-PY) were characterized using liquid chromatography coupled with quadrupole time-of-flight mass spectrometry (LC–QTOF–MS) following our previously established protocol [40]. The SNLM extract was dissolved in methanol and filtered through a 0.2 µm polytetrafluoroethylene (PTFE) syringe filter (Cytiva, Marlborough, MA, USA) prior to analysis. Samples were injected into the LC–QTOF–MS system. The chromatographic separation was performed on an Agilent 1290 Infinity LC system (Agilent Technologies, Santa Clara, CA, USA) using a Poroshell 120 EC-C18 column. For a chromatographic gradient, 0.1% formic acid in water and 0.1% formic acid in acetonitrile were used as the mobile phases for 33 min at 35 °C, with a flow rate of 200 µL/min. The LC unit was interfaced with an Agilent 6540 QTOF mass spectrometer fitted with an electrospray ionization (ESI) source and a diode-array detector. Mass spectra were acquired in both positive and negative ionization modes. Raw data were analyzed using Agilent MassHunter Workstation software (Qualitative Analysis, version B.08.00). Putative metabolite identification was proposed based on mass measurements, isotopic distribution, mass error values, and MS/MS fragmentation behavior. The proposed identities were further verified through comparison with entries in the Agilent Personal Compound Database and Library (PCDL), as well as published literature and publicly available metabolite databases containing compounds previously reported in *S. nervosum*.

### 4.7. DPPH Assay

The antioxidant activity of the SNLM extracts was evaluated using the DPPH radical scavenging assay as previously described [40]. The percentage of DPPH scavenging activity was calculated. The IC_50_ value was obtained from concentration–response curves and expressed as the extract concentration required to inhibit 50% of DPPH radicals.

### 4.8. ABTS Assay

The antioxidant activity of the SNLM extracts was evaluated using the ABTS radical cation decolorization assay as previously described [40]. The IC_50_ value was obtained from concentration–response curves and expressed as the extract concentration required to inhibit 50% of DPPH radicals.

### 4.9. Ferric Reducing Antioxidant Power (FRAP) Assay

The ferric reducing antioxidant power (FRAP) of the SNLM extracts was determined according to a previously described protocol [40]. The antioxidant capacity of the extracts was calculated from the calibration curve. The results were expressed as mmol TE/g extract.

### 4.10. Oxygen Radical Absorbance Capacity (ORAC) Assay

The ORAC assay was determined according to a previously described protocol with minor modification [41]. Briefly, SNLM extracts were mixed with fluorescein solution in a black 96-well plate and preincubated at 37 °C for 30 min. After that, 2,2-azobis(2-amidinopropane) dihydrochloride (AAPH), a peroxyl radical generator, was added to all wells, and the plate was incubated at 37 °C. The fluorescence signal was measured every minute for 2 h using a fluorescence microplate reader (excitation wavelength: 485 nm; emission wavelength: 525 nm). The antioxidant capacity of the SNLM extracts was determined by calculating the area under the fluorescence decay curve (AUC). The results were expressed as μmol TE/g sample.

### 4.11. Anti-Tyrosinase Activity

The anti-tyrosinase activity was determined according to a previously described protocol with some modifications [42]. Briefly, kojic acid (Sigma-Aldrich, St. Louis, MO, USA) solution (0–1000 µg/mL) was used as the positive control, and SNLM extracts (0–20 mg/mL) were mixed with 1X phosphate buffer and incubated at 37 °C for 10 min. Subsequently, mushroom tyrosinase enzyme solution (100 Units/mL) was added, and the mixture was further incubated at 37 °C for 20 min. After the incubation period, 2.5 mM L-DOPA was added as the substrate. The absorbance was then measured at 490 nm. The percentage of inhibition was calculated using the following equation:

% Inhibition = [(A − B) − (C − D)/(A − B)] × 100

where

A = Absorbance of the negative control solution containing phosphate buffer and enzyme

B = Absorbance of the blank for the negative control

C = Absorbance of the sample solution containing the test sample and the enzyme

D = Absorbance of the blank for the sample solution

The IC_50_ value, defined as the concentration of extract required to achieve 50% inhibition of tyrosinase activity, was determined from the dose–response curve and expressed in mg/mL.

### 4.12. Bacterial Strains

Four bacterial reference strains were used to evaluate the antibacterial activity of the extract. *S. aureus* TISTR 746 and *S. epidermidis* TISTR 518 were selected as representatives of Gram-positive skin-associated pathogens, whereas *E. coli* TISTR 073 and *P. aeruginosa* TISTR 2730 were selected as representatives of Gram-negative bacteria frequently implicated in opportunistic or skin-related infections. All strains were maintained in Mueller–Hinton broth (MHB; HiMedia Laboratories, Mumbai, India) at 37 °C for 16–18 h. Prior to testing, cultures were streaked onto Mueller–Hinton agar (MHA) and incubated under identical conditions to obtain isolated colonies for subsequent experiments.

### 4.13. Determination of the Zone of Inhibition (ZOI)

Antibacterial activity was screened using the Kirby–Bauer disk diffusion method with minor modifications to standard protocols [24]. Briefly, a colony from a fresh culture was suspended in sterile 0.85% NaCl, and the turbidity was adjusted to a 0.5 McFarland standard (~1–2 × 10^8^ CFU/mL). A sterile cotton swab was immersed in the suspension and evenly spread across the entire surface of the MHA plates. Sterile paper disks were then placed onto the inoculated agar. The SNLM extract stock preparation was prepared by dissolving the dried extract in DMSO at a concentration of 500 mg/mL. The mixture was thoroughly vortexed to obtain a homogeneous preparation and filtered through a sterile syringe filter before use. For the disk diffusion assay, 10 µL of the stock preparation was applied to each sterile disk, yielding a final extract loading of 5 mg/disk. For the negative control, sterile disks were loaded with 10 µL of DMSO alone. Plates were incubated at 37 °C for 16–18 h. Following incubation, the diameter of the inhibition zone, including the disk, was measured in millimeters using a caliper. All experiments were performed in triplicate.

### 4.14. Determination of Minimum Inhibitory Concentration (MIC) and Minimum Bactericidal Concentration (MBC)

The MIC and MBC of the SNLM extract were determined using a broth microdilution method in 96-well microplates, following established procedures with minor modifications [43]. The bacterial inoculum was prepared from fresh cultures, adjusted to a final density of approximately 5 × 10^5^ CFU/mL per well, and mixed with 50 µL of SNLM-CM and SNLM-PY extracts at various concentrations, prepared in MHB medium. The plates were incubated at 37 °C for 24 h. After incubation, resazurin solution (1 mg/mL) was added to each well (10 µL), followed by an additional incubation for 4 h to observe color development. Wells showing no color change were considered indicative of bacterial growth inhibition and were recorded as the MIC. To determine the MBC, culture medium, 10 µL aliquots from wells showing no visible growth at or above the MIC were plated onto MHA and incubated at 37 °C for 24 h. The MBC was recorded as the lowest concentration that resulted in no colony formation on the agar surface. Chloramphenicol (MedChemExpress, Monmouth Junction, NJ, USA) was used as a positive control, whereas wells containing broth with solvent but without the extract served as negative controls. All experiments were performed in triplicate.

### 4.15. Time–Kill Kinetics Assay

The time–kill kinetics of *S. aureus* and *S. epidermidis* treated with SNLM extracts were evaluated using the method described by Shu et al. (2019) [44] with minor modifications. The extracts were prepared at final concentrations corresponding to 0.125×, 0.25×, 0.5×, and 1× MIC. A bacterial inoculum obtained from fresh cultures was adjusted to approximately 1 × 10^5^ CFU/mL. The assay was performed in 96-well microplates with a final volume of 100 µL per well. Chloramphenicol served as positive control, whereas DMSO without extract was used as the negative control at the same final concentration as that used in the extract-treated wells. Bacterial growth was monitored over a 24 h period at 37 °C. Optical density at 600 nm (OD_600_) was measured at 0, 1, 2, 3, 4, 8, and 24 h using a microplate reader.

### 4.16. Determination of the Bacterial Cell Viability

The effect of SNLM extracts on bacterial viability across different concentrations was evaluated using the PrestoBlue™ Cell Viability Reagent (Thermo Fisher Scientific, Waltham, MA, USA). Briefly, 50 µL of bacterial suspensions (approximately 1 × 10^5^ CFU/mL) were treated with either 50 µL of SNLM extracts (at concentrations of 0.125×, 0.25×, 0.5×, and 1× MIC). DMSO without extract was included as the vehicle control at the same final concentration as that used in the extract-treated wells. The plate was incubated for 24 h at 37 °C. Bacterial cell viability was determined using PrestoBlue™ Cell Viability Reagent. The absorbance was measured at 570 and 600 nm using a microplate reader. Bacterial viability was calculated relative to the untreated control according to the following equation: % Bacterial cell viability = [OD570-OD600) treated cells/(OD570-OD600) untreated cells] × 100.

### 4.17. Determination of the Bacterial Membrane Integrity

Bacterial viability was evaluated using a dual-fluorescence staining method based on membrane integrity, following the protocol of the Bacterial Viability Assay Kit (Abcam, Cambridge, UK). Briefly, isolates of *S. aureus* and *S. epidermidis* were streaked onto Mueller–Hinton agar (MHA) and incubated at 37 °C for 24 h to obtain isolated colonies. Bacterial suspensions were then prepared and adjusted to approximately 1 × 10^7^ CFU/mL. The SNLM extracts were prepared at a final concentration of 1× MIC. For each treatment, 500 µL of the bacterial suspension was mixed with 500 µL of the extract in microcentrifuge tubes and vortexed gently prior to incubation at 37 °C for 1 h. DMSO served as the negative control (at the same final concentration as in the extract-treated wells), whereas ampicillin served as the positive control. Following exposure, samples were centrifuged at 10,000× *g* for 10 min. The supernatant was removed, and the pellets were resuspended in 100 µL of 1× buffer containing the dead-cell and total-cell staining dyes. The mixtures were incubated in the dark at room temperature for 1 h. Fluorescence signals were measured using a fluorescence microplate reader with Excitation/emission wavelengths of 490 nm/525 nm (Reading 1) and 536 nm/617 nm (Reading 2). The percentage of dead cells was calculated using the following equation: % Dead cells = [Reading 2/Reading 1] × 100.

### 4.18. Measurement of Nucleic Acid and Protein Leakage

Bacterial cultures of *S. aureus* and *S. epidermidis* were prepared by adjusting cell suspensions to approximately 1 × 10^7^ CFU/mL. To evaluate nucleic acid and protein leakage as indicators of bacterial cell membrane damage, 500 µL aliquots of bacterial suspension were mixed with 500 µL of SNLM extracts to obtain a final concentration of 1× MIC in sterile microcentrifuge tubes. DMSO was employed as the negative control (at the same final concentration as in the extract-treated wells), whereas chloramphenicol served as the positive control. The tubes were gently inverted and incubated at 37 °C for 3 h. Following incubation, samples were centrifuged at 2500 rpm for 10 min at 25 °C. The supernatants were collected and filtered through a 0.22 µm syringe filter to remove residual cells and debris. The bacterial nucleic acid content was analyzed using a microspectrophotometer set to 260 nm, which estimates the amount of released nucleic acids in solution (indicative of compromised membrane integrity). Protein content was analyzed using the Bradford reagent (Bio-Rad Laboratories, Hercules, CA, USA). Briefly, the standard bovine serum albumin (BSA) solution was diluted in 1× PBS to concentrations ranging from 0 to 200 µg/mL. Subsequently, 200 µL of Bradford reagent was added to each well of a 96-well plate, followed by 20 µL of the prepared BSA standard solutions or filtered samples. The mixtures were gently mixed and incubated for 10 min. The absorbance was then measured at 600 nm. A standard curve was plotted with absorbance values against protein concentrations. The obtained absorbance values were compared with the BSA standard curve to determine the protein concentration of the samples.

### 4.19. Protein Profile Analysis by Sodium Dodecyl Sulfate–Polyacrylamide Gel Electrophoresis (SDS–PAGE)

Protein profile patterns of *S. aureus* and *S. epidermidis* following exposure to SNLM extracts were analyzed by SDS–PAGE. Bacterial suspensions were prepared at approximately 1 × 10^7^ CFU/mL. The extract was added to the cultures at a final concentration equivalent to 1× MIC. Briefly, 2.5 mL of the extract solution was mixed with 2.5 mL of the bacterial suspension and incubated at 37 °C for 48 h. After treatment, cells were harvested by centrifugation at 10,000× *g* for 10 min. The resulting pellets were resuspended in lysis buffer and incubated on ice for 30 min to lyse bacterial membranes. The lysates were further centrifuged at 13,000× *g* for 15 min at 4 °C to remove cell debris. The supernatants containing soluble bacterial proteins were collected, mixed with 5× loading dye, and heated at 95 °C for 5 min. Protein samples were then separated by 10% SDS–PAGE. After electrophoresis, gels were stained overnight with Coomassie Brilliant Blue R-250 and subsequently destained until protein bands were clearly visible. The stained gel was captured by the Gel Doc XR+ Gel Documentation System (Bio-Rad Laboratories, Hercules, CA, USA).

### 4.20. Scanning Electron Microscopy (SEM) Analysis

Morphological alterations of *S. aureus* and *S. epidermidis* after exposure to the SNLM extract were examined by SEM. Bacterial suspensions were treated with the extract at a concentration equivalent to 1× MIC and incubated at 37 °C for 3 h. Following treatment, cells were collected by centrifugation at 10,000 rpm for 5 min, washed with 1× PBS, and placed onto 0.2 µm membrane filters. The samples were air-dried and subsequently washed with 0.1 M phosphate buffer before post-fixation with 1% osmium tetroxide (OsO_4_) for 1 h. The specimens were dehydrated through a graded ethanol series (50%, 70%, 90%, and 100%), allowing 10 min at each step, followed by immersion in absolute ethanol for 20 min. Samples were dried using a critical-point dryer, mounted on stubs, and sputter-coated with gold. The prepared samples were then observed under a scanning transmission electron microscope (SEM) (JEOL, Tokyo, Japan).

### 4.21. Cytotoxicity Assay Against Normal Human Dermal Fibroblasts (NHDF)

The cytotoxicity of the SNLM extracts toward normal human dermal fibroblasts (NHDF) was assessed using an MTT assay. Briefly, NHDF cells were plated into 96-well plates at a density of 5 × 10^3^ cells per well and allowed to attach prior to treatment. The cells were then exposed to SNLM extracts at concentrations ranging from 0 to 800 µg/mL and incubated at 37 °C with 5% CO_2_ for 24, 48, and 72 h. Cell viability was evaluated using the MTT solution. Absorbance was subsequently measured at 570 nm using a microplate reader. Cell viability was expressed as a percentage relative to untreated controls.

### 4.22. Preparation of Cleansing Gel Base

Initially, purified water (56.10% *w*/*w*) was heated to approximately 70 °C and stirred at 300 rpm using an overhead stirrer (IKA-Werke GmbH & Co. KG, Staufen, Germany). PEG-150 Distearate (0.3% *w*/*w*) was then added as a thickening agent and mixed continuously until completely dissolved. Subsequently, the surfactants, including Plantacare (10% *w*/*w*), Disodium Laureth Sulfosuccinate (20% *w*/*w*), Cocamidopropyl Betaine (3.5% *w*/*w*), PEG-7 Glyceryl Cocoate (4.5% *w*/*w*), and PEG-RH-40 (2% *w*/*w*), were gradually incorporated under gentle continuous stirring to minimize foam formation and ensure homogeneity of the mixture. Thereafter, glycerine (2% *w*/*w*) and allantoin (0.3% *w*/*w*) were added as moisturizing and skin-soothing agents, respectively, and the formulation was mixed thoroughly until a smooth, homogeneous gel was achieved. The mixture was then cooled to below 40 °C before adding phenoxyethanol (0.8% *w*/*w*) and perfume (0.5% *w*/*w*) to preserve their stability and fragrance quality. Finally, the formulation was continuously stirred until a clear and homogeneous shower gel was obtained.

### 4.23. SNLM Cleansing Gel Formulations

The SNLM extract solutions were prepared by dissolving 2 g of the crude extract into 8 g of 95% ethanol to obtain a 20% (*w*/*w*) extract solution. The mixtures were stirred continuously until the extracts were completely dissolved and homogeneous solutions were obtained. To prepare cleansing gel samples, the SNLM extract solutions were incorporated into the gel base at varying concentrations. The 1× formulation was prepared by mixing 0.65 g of the 20% extract solution with 9.35 g of gel base, yielding a total weight of 10 g. Similarly, the 2.5× formulation was prepared by incorporating 1.66 g of the 20% extract solution into 8.34 g of gel base, resulting in a final weight of 10 g. SNLM cleansing gel formulations were thoroughly mixed by gentle stirring until uniform, yielding homogeneous products. The prepared samples were then stored in tightly closed containers at room temperature. SNLM-CM and SNLM-PY cleansing gel samples were evaluated for antibacterial activity using the zone of inhibition (ZOI) method, as described in 4.13.

### 4.24. Statistical Analysis

All experiments were performed with at least three independent replicates. Data are presented as mean ± standard error of the mean (SEM). Comparisons between two independent groups were analyzed using an unpaired Student’s *t*-test. For comparisons among multiple groups, statistical significance was determined using one-way or two-way analysis of variance (ANOVA), followed by Tukey’s multiple comparison test. A *p*-value of <0.05 was considered statistically significant. All analyses were carried out using GraphPad Prism version 8.

## 5. Conclusions

In conclusion, *S. nervosum* leaf extracts demonstrated promising antibacterial, antioxidant, and anti-tyrosinase activities relevant to skin health applications. The extracts were effective against key skin-associated bacteria, particularly *S. aureus* and *S. epidermidis*, with evidence suggesting membrane disruption as a primary antibacterial mechanism. Variations between SNLM-CM and SNLM-PY indicate that geographical origin influences phytochemical composition and bioactivity. The strong antioxidant capacity, together with enzyme inhibition, supports their potential to reduce oxidative stress and skin-related processes. Importantly, the extracts exhibited acceptable cytotoxicity against normal skin fibroblasts at appropriate concentrations and retained their activity when incorporated into a cleansing gel formulation. Overall, these findings highlight the potential of *S. nervosum* leaf extracts as multifunctional natural ingredients for dermatological and cosmetic applications. However, further studies are needed to confirm their efficacy and safety *in vivo*.

## Figures and Tables

**Table 1:** Percentage yield (%), TPC, and TFC of SNLM extracts.

Extract Samples	Initial Weight (g)	Weight of Extract (g)	%Yield	TPC (mg GAE/g Sample)	TFC (mg CE/g Sample)
SNLM-CM	800	36.37	4.55	167.48 ± 3.66	ND ^1^
SNLM-PY	700	45.10	6.44	179.82 ± 6.26 *	ND ^1^

Results are presented as the mean ± standard error of the mean (SEM) from three independent experiments (N = 3). The asterisk (*) indicates a significant difference between SNLM-CM and SNLM-PY (*p* < 0.05), as determined by an unpaired Student’s *t*-test. ^1^ ND, not detected; GAE, gallic acid equivalent; CE, catechin equivalent.

**Table 2:** Phytochemical profile of compounds identified in negative and positive electrospray ionization (ESI) modes in both SNLM-CM and SNLM-PY extracts by LC–QTOF–MS.

No.	RT (min)	Mass	*m*/*z* (Expected)	Chemical Formula	Identification Compounds
1	5.333	170.0211	169.0138	C_7_H_6_O_5_	gallic acid
2	7.461	198.0528	199.0593	C_9_H_10_O_5_	4-hydroxy-3,5-dimethoxybenzoic acid
3	7.604	306.0749	307.0824	C_15_H_14_O_7_	(-)-epigallocatechin
4	9.260	138.0319	183.0301	C_7_H_6_O_3_	4-hydroxybenzoic acid
5	9.260	184.0374	183.0301	C_8_H_8_O_5_	methyl-3,4,5-trihydroxybenzoate
6	12.678	302.0065	300.999	C_14_H_6_O_8_	ellagic acid
7	12.790	166.0629	165.0556	C_9_H_10_O_3_	3-hydroxy-3-phenylpropanoic acid
8	16.662	434.0859	433.0787	C_20_H_18_O_11_	reynoutrin
9	16.833	462.0811	461.0734	C_21_H_18_O_12_	3-O-Methyl ellagic acid-4′-*O*-a-L-rhamnopyranoside
10	18.540	164.0834	223.0974	C_10_H_12_O_2_	eugenol
11	19.053	344.0535	343.0464	C_17_H_12_O_8_	3,3′,4′-tri-*O*-methylellagic acid
12	19.281	206.0951	251.0932	C_12_H_14_O_3_	eugenyl acetate
13	19.679	300.1000	299.0928	C_17_H_16_O_5_	(2S,3S)-2,3-trans-5,7-dihydroxy-6,8-dimethyldihydroflavonol
14	19.736	488.3511	487.3438	C_30_H_48_O_5_	arjunolic acid
15	20.078	486.3352	485.3276	C_30_H_46_O_5_	actinidic acid
16	20.819	312.1009	311.0937	C_18_H_16_O_5_	(2S)-8-formyl-5-hydroxy-7-methoxy-6-methylflavanone
17	21.104	618.3928	663.3913	C_39_H_54_O_6_	3-*O*-cis-*p*-coumaroylmaslinic acid
18	21.730	450.2411	449.2341	C_28_H_34_O_5_	cleistocaltone B
19	21.787	472.3557	471.3484	C_30_H_48_O_4_	2-hydroxyursolic acid
20	23.895	456.3611	455.3538	C_30_H_48_O_3_	ursolic acid

**Table 3:** Screening of antibacterial activity of SNLM extracts against *S. aureus*, *S. epidermidis*, *E. coli*, and *P. aeruginosa*.

Extract Samples	*S. aureus* TISTR 746			*S. epidermis* TISTR 518			*E. coli* TISTR 073			*P. aeruginosa* TISTR 2730		
	ZOI (mm)	MIC (mg/mL)	MBC (mg/mL)	ZOI (mm)	MIC (mg/mL)	MBC (mg/mL)	ZOI (mm)	MIC (mg/mL)	MBC (mg/mL)	ZOI (mm)	MIC (mg/mL)	MBC (mg/mL)
SNLM-CM	20.00 ± 2.00	3.26 ± 1.13	6.52 ± 2.26	23.33 ± 2.08	5.21 ± 2.26	9.12 ± 5.97	7.00 ± 1.73	15.63 ± 0.00	31.25 ± 0.00	14.33 ± 1.15	10.42 ± 4.51	20.84 ± 9.02
SNLM-PY	11.67 ± 0.58	6.52 ± 2.26	13.03 ± 4.51	17.67 ± 1.53	3.26 ± 1.13	6.52 ± 2.26	6.17 ± 0.29	31.25 ± 0.00	65.50 ± 0.00	9.67 ± 1.15	15.63 ± 0.00	31.25 ± 0.00

Results are presented as the mean ± SEM from three independent experiments (N = 3).

**Table 4:** Antioxidant and anti-tyrosinase activities of *S. nervosum* leaf methanolic (SNLM) extracts.

Extract Samples	IC_50_ ABTS (µg/mL)	IC_50_ DPPH (µg/mL)	FRAP (mM TE/g Sample)	ORAC (µM TE/mg Sample)	IC_50_ Anti-Tyrosinase Activity (mg/mL)
SNLM-CM	8.00 ± 2.00	16.00 ± 1.79 ***	1525.71 ± 24.62	997.05 ± 40.80	5.61 ± 0.24
SNLM-PY	11.00 ± 1.44 *	11.00 ± 1.12 **	1306.79 ± 49.75	750.29 ± 22.50	1.28 ± 0.28
Trolox	6.95 ± 0.24	6.40 ± 0.05	ND ^2^	ND ^2^	ND ^2^
Kojic acid	ND ^2^	ND ^2^	ND ^2^	ND ^2^	0.118 ± 0.01

Results are presented as the mean ± SEM from three independent experiments (N = 3). Trolox was used as the positive control for the ABTS and DPPH antioxidant assays, whereas kojic acid served as the positive control for the anti-tyrosinase activity assay. The asterisks (* *p* < 0.05, ** *p* < 0.01, and *** *p* < 0.001) indicate significant differences between SNLM-CM or SNLM-PY and Trolox, as determined by one-way ANOVA followed by Tukey’s multiple comparison test. ^2^ ND, not determined; TE, Trolox equivalent.

## Data Availability

All data generated or analyzed in this study are included in this article and its Supplementary Materials.

## Supplementary Materials

The following supporting information can be downloaded at: https://www.mdpi.com/article/10.3390/ijms27146515/s1.

## Abbreviations

The following abbreviations are used in this manuscript:

CHL	Chloramphenicol
DMSO	Dimethyl sulfoxide
FRAP	Ferric reducing antioxidant power
GAE	Gallic acid equivalent
MBC	Minimum bactericidal concentration
MIC	Minimum inhibitory concentration
*m*/*z*	Mass-to-charge ratio
NHDF	Normal human dermal fibroblasts
ORAC	Oxygen radical absorbance capacity
RT	Retention time
SNLM	*Syzygium nervosum* leaf methanolic extract
SNLM-CM	*Syzygium nervosum* leaf methanolic extract from Chiang Mai
SNLM-PY	*Syzygium nervosum* leaf methanolic extract from Phayao
TE	Trolox equivalent
TFC	Total flavonoid content
TPC	Total phenolic content
ZOI	Zone of inhibition

## References

[1] Grice E.A., Segre J.A. (2011). The skin microbiome. Nat. Rev. Microbiol..

[2] Tong S.Y., Davis J.S., Eichenberger E., Holland T.L., Fowler V.G. (2015). Staphylococcus aureus infections: Epidemiology, pathophysiology, clinical manifestations, and management. Clin. Microbiol. Rev..

[3] Otto M. (2009). Staphylococcus epidermidis--the ‘accidental’ pathogen. Nat. Rev. Microbiol..

[4] Brown A.F., Leech J.M., Rogers T.R., McLoughlin R.M. (2014). Staphylococcus aureus Colonization: Modulation of Host Immune Response and Impact on Human Vaccine Design. Front. Immunol..

[5] Davies J., Davies D. (2010). Origins and evolution of antibiotic resistance. Microbiol. Mol. Biol. Rev..

[6] Rinnerthaler M., Bischof J., Streubel M.K., Trost A., Richter K. (2015). Oxidative stress in aging human skin. Biomolecules.

[7] Mani Iyer P., Sivamaruthi B., Sukprasansap M., Chuchawankul S., Tencomnao T., Chaiyasut C. (2020). Functional properties and Bioactivities of *Cleistocalyx nervosum* var. paniala berry plant: A review. Food Sci. Technol..

[8] Pham G.N., Nguyen-Ngoc H. (2020). Ethnopharmacology, Phytochemistry, and Pharmacology of *Syzygium nervosum*. Evid.-Based Complement. Altern. Med..

[9] Dung N.T., Kim J.M., Kang S.C. (2008). Chemical composition, antimicrobial and antioxidant activities of the essential oil and the ethanol extract of *Cleistocalyx operculatus* (Roxb.) Merr and Perry buds. Food Chem. Toxicol..

[10] Thanh D.T., Oanh V.K., Nguyen H.C., Ngan L.T.M., Hieu T.T. (2024). Phytochemical composition, antioxidant, antibacterial, and enzyme inhibitory activities of organic extracts from flower buds of *Cleistocalyx operculatus* (Roxb.) Merr. et Perry. BioTechnologia.

[11] Nakatsuji T., Chen T.H., Butcher A.M., Trzoss L.L., Nam S.J., Shirakawa K.T., Zhou W., Oh J., Otto M., Fenical W. (2018). A commensal strain of *Staphylococcus epidermidis* protects against skin neoplasia. Sci. Adv..

[12] Pan L., Yang N., Sui Y., Li Y., Zhao W., Zhang L., Mu L., Tang Z. (2023). Altitudinal Variation on Metabolites, Elements, and Antioxidant Activities of Medicinal Plant Asarum. Metabolites.

[13] Bai J., Zhang Y., Tang C., Hou Y., Ai X., Chen X., Zhang Y., Wang X., Meng X. (2021). Gallic acid: Pharmacological activities and molecular mechanisms involved in inflammation-related diseases. Biomed. Pharmacother..

[14] Alruhaimi R., Hassanein E., Althagafy H., El Mohtadi M., Mahmoud A. (2026). Reynoutrin Mitigates Metabolic, Immune, and Redox Dysregulation in Acute Dyslipidemia. Kafkas Univ. Vet. Fak. Derg..

[15] Saraphanchotiwitthaya A., Khorana N., Sripalakit P. (2019). Comparative anti-inflammatory activity of eugenol and eugenyl acetate on the murine immune response in vitro. Songklanakarin J. Sci. Technol..

[16] Hemalatha T., Pulavendran S., Balachandran C., Manohar B.M., Puvanakrishnan R. (2010). Arjunolic acid: A novel phytomedicine with multifunctional therapeutic applications. Indian J. Exp. Biol..

[17] Song J.-G., Su J.-C., Song Q.-Y., Huang R.-L., Tang W., Hu L.-J., Huang X.-J., Jiang R.-W., Li Y.-L., Ye W.-C. (2019). Cleistocaltones A and B, Antiviral Phloroglucinol–Terpenoid Adducts from Cleistocalyx operculatus. Org. Lett..

[18] Liu G., Qin P., Cheng X., Wu L., Wang R., Gao W. (2023). Ursolic acid: Biological functions and application in animal husbandry. Front. Vet. Sci..

[19] Ncube B., Finnie J.F., Van Staden J. (2012). Quality from the field: The impact of environmental factors as quality determinants in medicinal plants. S. Afr. J. Bot..

[20] Pandey K.B., Rizvi S.I. (2009). Plant polyphenols as dietary antioxidants in human health and disease. Oxid. Med. Cell Longev..

[21] Dai J., Mumper R.J. (2010). Plant Phenolics: Extraction, Analysis and Their Antioxidant and Anticancer Properties. Molecules.

[22] Sampaio B.L., Edrada-Ebel R., Da Costa F.B. (2016). Effect of the environment on the secondary metabolic profile of *Tithonia diversifolia*: A model for environmental metabolomics of plants. Sci. Rep..

[23] Sari K.R.P., Ikawati Z., Danarti R., Hertiani T. (2023). Micro-titer plate assay for measurement of total phenolic and total flavonoid contents in medicinal plant extracts. Arab. J. Chem..

[24] Do Q.D., Angkawijaya A.E., Tran-Nguyen P.L., Huynh L.H., Soetaredjo F.E., Ismadji S., Ju Y.-H. (2014). Effect of extraction solvent on total phenol content, total flavonoid content, and antioxidant activity of Limnophila aromatica. J. Food Drug Anal..

[25] Takó M., Kerekes E.B., Zambrano C., Kotogán A., Papp T., Krisch J., Vágvölgyi C. (2020). Plant Phenolics and Phenolic-Enriched Extracts as Antimicrobial Agents against Food-Contaminating Microorganisms. Antioxidants.

[26] Miklasińska-Majdanik M., Kępa M., Wojtyczka R.D., Idzik D., Wąsik T.J. (2018). Phenolic Compounds Diminish Antibiotic Resistance of *Staphylococcus aureus* Clinical Strains. Int. J. Environ. Res. Public Health.

[27] Singh G., Passsari A.K., Leo V.V., Mishra V.K., Subbarayan S., Singh B.P., Kumar B., Kumar S., Gupta V.K., Lalhlenmawia H. (2016). Evaluation of Phenolic Content Variability along with Antioxidant, Antimicrobial, and Cytotoxic Potential of Selected Traditional Medicinal Plants from India. Front. Plant Sci..

[28] Zacchino S.A., Butassi E., Liberto M.D., Raimondi M., Postigo A., Sortino M. (2017). Plant phenolics and terpenoids as adjuvants of antibacterial and antifungal drugs. Phytomedicine.

[29] Apak R., Özyürek M., Güçlü K., Çapanoğlu E. (2016). Antioxidant Activity/Capacity Measurement. 1. Classification, Physicochemical Principles, Mechanisms, and Electron Transfer (ET)-Based Assays. J. Agric. Food Chem..

[30] Shah P., Modi H. (2015). Comparative Study of DPPH, ABTS and FRAP Assays for Determination of Antioxidant Activity. Int. J. Res. Appl. Sci. Eng. Technol..

[31] Prior R.L., Wu X., Schaich K. (2005). Standardized methods for the determination of antioxidant capacity and phenolics in foods and dietary supplements. J. Agric. Food Chem..

[32] Alam M.N., Bristi N.J., Rafiquzzaman M. (2013). Review on in vivo and in vitro methods evaluation of antioxidant activity. Saudi Pharm. J..

[33] Kubglomsong S., Theerakulkait C., Reed R.L., Yang L., Maier C.S., Stevens J.F. (2018). Isolation and Identification of Tyrosinase-Inhibitory and Copper-Chelating Peptides from Hydrolyzed Rice-Bran-Derived Albumin. J. Agric. Food Chem..

[34] Chang T.-S. (2012). Natural Melanogenesis Inhibitors Acting Through the Down-Regulation of Tyrosinase Activity. Materials.

[35] Zolghadri S., Bahrami A., Hassan Khan M.T., Munoz-Munoz J., Garcia-Molina F., Garcia-Canovas F., Saboury A.A. (2019). A comprehensive review on tyrosinase inhibitors. J. Enzym. Inhib. Med. Chem..

[36] Gavanji S., Bakhtari A., Famurewa A.C., Othman E.M. (2023). Cytotoxic Activity of Herbal Medicines as Assessed in Vitro: A Review. Chem. Biodivers..

[37] Ghuman S., Ncube B., Finnie J.F., McGaw L.J., Coopoosamy R.M., Van Staden J. (2016). Antimicrobial Activity, Phenolic Content, and Cytotoxicity of Medicinal Plant Extracts Used for Treating Dermatological Diseases and Wound Healing in KwaZulu-Natal, South Africa. Front. Pharmacol..

[38] Mukherjee P.K., Maity N., Nema N.K., Sarkar B.K. (2011). Bioactive compounds from natural resources against skin aging. Phytomedicine.

[39] Daglia M. (2012). Polyphenols as antimicrobial agents. Curr. Opin. Biotechnol..

[40] Thepmalee C., Nuntaboon P., Songkrao A., Onsa-Ard A., Suwannasom N., Khoothiam K., Prommana R., Wonghempoom A., Thim-Uam A. (2026). Phytochemical profiling of *Syzygium nervosum* fruit pulp extracts and their modulatory effects on oxidative stress and cytokine production. BMC Complement. Med. Ther..

[41] Thaipong K., Boonprakob U., Crosby K., Cisneros-Zevallos L., Hawkins Byrne D. (2006). Comparison of ABTS, DPPH, FRAP, and ORAC assays for estimating antioxidant activity from guava fruit extracts. J. Food Compos. Anal..

[42] Cui H.-X., Duan F.-F., Jia S.-S., Cheng F.-R., Yuan K. (2018). Antioxidant and Tyrosinase Inhibitory Activities of Seed Oils from *Torreya grandis* Fort. ex Lindl. BioMed. Res. Int. BioMed..

[43] Wiegand I., Hilpert K., Hancock R.E. (2008). Agar and broth dilution methods to determine the minimal inhibitory concentration (MIC) of antimicrobial substances. Nat. Protoc..

[44] Shu Q., Niu Y., Zhao W., Chen Q. (2019). Antibacterial activity and mannosylerythritol lipids against vegetative cells and spores of *Bacillus cereus*. Food Control.

