# Supplementary material for: Antibacterial Mechanisms and Skin-Protective Activities of Syzygium nervosum Leaf Extracts from Different Geographical Origins
Source: Int J Mol Sci. 2026 Jul 22;27(14):6515. doi: 10.3390/ijms27146515 (PMC13411812; doi:10.3390/ijms27146515)
Supplement: Supplementary file 1 [file ijms-27-06515-s001.zip › ijms-4383356-supplementary.pdf]

Supplementary Materials

Table S1 Phytochemical profile of compounds identified in negative and positive electrospray ionization (ESI) modes in SNLM-CM extract by LC–QTOF-MS.

No	RT (min)	Mass	m/z (Expected)	Chemical Formula	Identification compounds
1	19.746	472.3566	473.3637	$C_{30}H_{48}O_4$	2-hydroxyursolic acid
2	22.107	300.1375	301.1448	$C_{18}H_{20}O_4$	2'-hydroxy-4',6'-dimethoxy-3'-methyldihydrochalcone
3	8.748	314.0782	359.0763	$C_{17}H_{14}O_6$	(2S)-8-formyl-6-methylnaringenin
4	11.367	346.0674	405.0812	$C_{17}H_{14}O_8$	5,7,8,4'-tetrahydroxy-3',5'-dimethoxyflavone-3-O-b-D-galactopyranoside
5	13.360	286.1208	331.1190	$C_{17}H_{18}O_4$	myrigalone-G
6	13.986	478.1119	477.1045	$C_{22}H_{22}O_{12}$	mearnsitrin
7	15.068	302.0427	301.0358	$C_{15}H_{10}O_7$	quercetin
8	15.181	286.0482	331.0462	$C_{15}H_{10}O_6$	kaernpferol
9	15.978	270.0897	315.0878	$C_{16}H_{14}O_4$	2',4'-dihydroxy-6'-methoxchalcone
10	16.890	316.0587	315.0517	$C_{16}H_{12}O_7$	tamarixetin
11	17.687	274.084	333.0984	$C_{15}H_{14}O_5$	phloretin
12	19.110	504.3457	503.3383	$C_{30}H_{48}O_6$	2,3,6,23-tetrahydroxyurs-12-en-28-oic acid
13	20.021	296.1053	295.098	$C_{18}H_{16}O_4$	7-hydroxy-5-methoxy-6,8-dimethylflavone
14	20.420	448.2251	447.2185	$C_{28}H_{32}O_5$	cleistocaltone A
15	20.534	314.1165	313.1093	$C_{18}H_{18}O_5$	2,2',4'-trihydroxy-6'-methoxy-3',5'-dimethylchalcone
16	20.762	284.1058	283.0986	$C_{17}H_{16}O_4$	(2S)-5,7-dihydroxy-6,8-dimethylflavanone
17	20.933	470.3409	529.3545	$C_{30}H_{46}O_4$	cleistocalyxic acid A
18	21.559	298.1209	297.1134	$C_{18}H_{18}O_4$	(2S)-5-hydroxy-7-methoxy-6,8-dimethylflavanone

RT, retention time (minutes); m/z, mass-to-charge ratio

Table S2 Phytochemical profile of compounds identified in negative and positive electrospray ionization (ESI) modes in SNLM-PY extract by LC–QTOF-MS.

No	RT (min)	Mass	m/z (Expected)	Chemical Formula	Identification compounds
1	1.776	148.0525	166.0863	$C_9H_8O_2$	cinnamic acid
2	1.842	458.0859	459.0932	$C_{22}H_{18}O_{11}$	(-)-epigallocatechin 3-O-gallate
3	3.854	354.0958	355.1032	$C_{16}H_{18}O_9$	isobiflorin
4	12.111	490.1491	491.1568	$C_{24}H_{26}O_{11}$	3'formyl-6',4-dihydroxy-2'-methoxy-5'-methylchalcone-4'-*O*-b-D-glucopyranoside
5	12.246	206.0947	224.1288	$C_{12}H_{14}O_3$	eugenyl acetate
6	15.997	346.0675	347.0748	$C_{17}H_{14}O_8$	5,7,8,4'-tetrahydroxy-3',5'-dimethoxyflavone-3-*O*-b-D-galactopyranoside
7	16.344	940.1199	958.1551	$C_{41}H_{32}O_{26}$	1,2,3,4,6-penta-*O*-galloyl-b-d-glucose
8	20.093	426.3878	449.3775	$C_{30}H_{50}O$	lupeol
9	8.351	444.2001	443.1927	$C_{21}H_{32}O_{10}$	4'-dihydrophaseic acid
10	13.191	494.0678	493.061	$C_{21}H_{18}O_{14}$	myricetin-3-O-glucoronide
11	13.363	480.0905	479.0830	$C_{21}H_{20}O_{13}$	myricetin-3-*O*-glucoside
12	13.647	198.0532	197.0459	$C_9H_{10}O_5$	4-hydroxy-3,5-dimethoxybenzoic acid
13	13.874	122.0363	167.0346	$C_7H_6O_2$	hydroxybenzaldehyde
14	16.380	940.1195	939.1126	$C_{41}H_{32}O_{26}$	1,2,3,4,6-penta-O-galloyl-b-d-glucose
15	16.661	520.122	579.1357	$C_{24}H_{24}O_{13}$	mearnsetin 3-O-(4''-O-acety)-a-l-rhamnopyranoside
16	17.519	658.1168	657.1086	$C_{30}H_{26}O_{17}$	myricetrin 4''-*O*-acetyl-2''-*O*-gallate
17	19.114	518.3619	517.3544	$C_{31}H_{50}O_6$	cleistocalyxic acid F
18	22.132	618.3947	617.3874	$C_{39}H_{54}O_6$	3-*O*-cis-*p*-coumaroylmaslinic acid
19	25.257	424.2628	423.2555	$C_{27}H_{36}O_4$	jambone F

RT, retention time (minutes); m/z, mass-to-charge ratio
